# Salt tolerance research in date palm tree (*Phoenix dactylifera* L.), past, present, and future perspectives

**DOI:** 10.3389/fpls.2015.00348

**Published:** 2015-05-18

**Authors:** Mahmoud W. Yaish, Prakash P. Kumar

**Affiliations:** ^1^Department of Biology, College of Science, Sultan Qaboos University, Muscat, Oman; ^2^Department of Biological Sciences, National University of Singapore, Singapore, Singapore

**Keywords:** salinity, date palm, omics, abiotic stress, reverse genetics, *Phoenix dactylifera* L.

## Abstract

The date palm can adapt to extreme drought, to heat, and to relatively high levels of soil salinity. However, excessive amounts of salt due to irrigation with brackish water lead to a significant reduction in the productivity of the fruits as well as marked decrease in the viable numbers of the date palm trees. It is imperative that the nature of the existing salt-adaptation mechanism be understood in order to develop future date palm varieties that can tolerate excessive soil salinity. In this perspective article, several research strategies, obstacles, and precautions are discussed in light of recent advancements accomplished in this field and the properties of this species. In addition to a physiological characterization, we propose the use of a full range of OMICS technologies, coupled with reverse genetics approaches, aimed toward understanding the salt-adaption mechanism in the date palm. Information generated by these analyses should highlight transcriptional and posttranscriptional modifications controlling the salt-adaptation mechanisms. As an extremophile with a natural tolerance for a wide range of abiotic stresses, the date palm may represent a treasure trove of novel genetic resources for salinity tolerance.

## Impact of Soil Salinity on Date Palm

It is widely believed that the date palm originated in the ancient Mesopotamia region or in western India (Wrigley, 1995). Currently, the date palm is the primary crop in several arid and semiarid countries in North Africa, the Middle East and Central America (Food and Agriculture Organization of the United Nations, 2006). For example, in Oman, the date palm occupies about 50% of the cultivated area and comprises 82% of all fruit crops grown in the country.

Salinity is the leading problem affecting agricultural production and ecosystems around the world. In recent decades, excessive soil salinity has become a global agricultural constraint (Rengasamy, 2006; Munns and Tester, 2008). This holds especially true in arid and semiarid regions where a considerable amount of agricultural land area has been affected (Pitman and Läuchli, 2002) and has led to significant economic losses in date palms and other crops (Cookson and Lepiece, 2001). This problem is more prevalent and the implications of the losses are more compelling in Oman and other Persian Gulf states due to the high evaporation rates from the soil surface (Stanger, 1985). The major causes of increased soil salinity in arid and semiarid regions are insufficient rainfall coupled with over-irrigation using brackish or saline groundwater (Hillel, 2000; Pitman and Läuchli, 2002; Malash et al., 2008). Soil salinity has resulted in desertification of large agricultural areas, particularly in coastal areas where the overharvesting of water has led to an infiltration of seawater into the groundwater (Stanger, 1985).

## Salt-Adaptation Capacity of the Date Palm

The date palm tree has evolved through natural selection to be a drought- and salt-tolerant plant (Zaid and de Wet, 2002a) with an adaptation capacity exceeding barley, which is widely considered to be a salt-tolerant crop (Furr and Armstrong, 1975). Some date palm varieties have the ability to grow in close proximity to the seashore where they are often exposed to seawater during tidal currents. Therefore, the date palm could be considered one of the exceptional halophytic plants and may possess a suite of mechanisms for salinity tolerance.

Plant species vary in their responses to saline conditions. While salt-tolerant plants have the ability to cope with a relatively high level of soil salinity, susceptible plants are not able to grow under the same salinity conditions (Munns and Tester, 2008). Soil salinity causes ionic toxicity and osmotic stress in salt-susceptible plants, a situation that reduces the growth rate and may lead to plant death. Very little scientific information is available regarding salt tolerance in the date palm. Ramoliya and Pandey (2003) screened particular date palm varieties for their salinity adaptive capacity and found that certain varieties can endure a relatively high soil salinity level of 12.8 dS m^–1^ (1 dS m^–1^ = 640 mg l^–1^) with no visible effect on the seedling phenotype. Another study conducted by Alrasbi et al. (2010) found that some other date palm varieties can tolerate up to 9 dS m^–1^ soil salinity, and an excess of Na^+^ ions accumulated in the leaves of the plants treated with high salt concentrations. Nevertheless, much work at the physiological and molecular levels is required in order to fully understand the salt-adaptation mechanisms in the date palm.

## What is Known about Salinity Adaptation Mechanisms in the Date Palm?

Plants can adapt to soil salinity through three major mechanisms: (i) osmotic tolerance, (ii) Na^+^ or Cl^–^ exclusion and secretion, and (iii) accumulation of Na^+^ or Cl^–^ in the tissues (Munns and Tester, 2008). These strategies involve different mechanisms, including the production of compatible solutes (Munns and Tester, 2008) such as sugars and sugar alcohols (e.g., trehalose, mannitol, and galactinol); the synthesis of amino acids and amines (e.g., proline and glycine betaine); the induction of free radical scavengers, such as superoxide dismutase (Hazman et al., 2015); the modulation of genes encoding transporters responsible for ionic balance, such as Na^+^/H^+^ antiporters and other Na^+^ transporters (Reguera et al., 2013), Na^+^ uniporters and/or ion channel-type transporters (Craig Plett and Moller, 2010; Assaha et al., 2015); the accumulation of late embryogenesis abundant (LEA) proteins (e.g., dehydrins; Hanin et al., 2011; Jyothi-Prakash et al., 2014); and the alteration of the hormonal contents of leaves and roots, such as abscisic acid (ABA; Mittler and Blumwald, 2015), indole acetic acid (IAA), and ethylene (Yaish et al., 2015). Additionally, plants trigger their epigenetic machinery to activate or deactivate various genes involved in salt tolerance, including miRNA biogenesis (Chaabane et al., 2012; Macovei and Tuteja, 2012) and DNA methylation processes (Tan, 2010; Jiang et al., 2014).

Despite the fact that molecular pathways underlying salt-adaptation mechanisms in some plant species have been dissected and partially identified, these mechanisms in the date palm still remain unknown. Previous work on salt-adaptation of date palm trees has focused on the genetic differentiation of adaptive varieties using DNA marker analysis, such as random amplified polymorphic DNA (RAPD; Sedra et al., 1998; Kurup et al., 2009) and microsatellites (Elshibli and Korpelainen, 2008). However, these analyses did not localize functional genes linked to salt-adaptation traits in this plant.

Additionally, work using date palm cell suspension cultures to investigate the response of palm cells to salinity has been recently reported (Al-Bahrany and Al-Khayri, 2012). The growth of these cells was negatively affected by salt treatment and the cellular Na^+^ content initially increased and then decreased within a few days of the treatment (Al-Bahrany and Al-Khayri, 2012). Furthermore, the proline levels were directly correlated with the NaCl concentration in the callus (Al-Khayri, 2002) and in the early-stage seedlings (Djibril et al., 2005). This type of study that is performed on cells or tissues generated *in vitro* can provide some information about the nature of salt-adaptation mechanisms. However, data from undifferentiated plant tissues lack the important functional input of various differentiated and well-developed tissues in the adaptation mechanism.

Recently, the date palm genome (*Khalas* variety) was sequenced (Al-Dous et al., 2011; Al-Mssallem et al., 2013), a genetic map was constructed (Mathew et al., 2014), and a group of conserved miRNA was computationally characterized (Xiao et al., 2013) based on the genome of the same variety. Moreover, gene expression analysis was performed on different plant tissues (Bourgis et al., 2011; Zhang et al., 2012); however, there is no gene expression or genetic marker data associated with salinity tolerance or any abiotic stress in date palms. In comparison with a few other date palm varieties, *Khalas* exhibited a higher level of salinity tolerance (Aljuburi, 1992; Al-Mulla et al., 2013). Therefore, it might be used as a standard variety by the research community given that the information generated by OMICS analysis, using this variety, has some advantages in salinity tolerance research. However, it is difficult to know if *Khalas* is the most tolerant among the other varieties because a comprehensive study screening a large number of date palm varieties for their salinity tolerance is still unavailable.

## How Can the Salinity-Adaptation Mechanism in the Date Palm be Deciphered?

Although date palms have the ability to grow under saline conditions, different varieties have displayed distinct levels of tolerance to soil salinity. Indigenous varieties growing in relatively saline soils and showing a normal phenotype may possess genes responsible for a more efficient salinity adaption mechanism than those growing in less saline soils. However, the question remains as to how these plants are able to grow in such a condition while others cannot. Do they exclude, compartmentalize, or secrete the NaCl? Do their tissues possess unique osmotic tolerance mechanisms? In order to find answers for these questions, varieties of date palms growing around the world should be characterized. A starting point would be to perform essential physiological and morphological characterizations (including growth rates, histology, tissue content, ion transport, etc.; Yeo et al., 1999; Gong et al., 2006; Faiyue et al., 2012). From these, a small group of extremely salt-tolerant and salt-susceptible varieties should be studied in depth. It is known that some other salt-tolerant species, such as mangroves, develop specific apoplastic barriers that can act as filters against NaCl uptake in their roots, minimizing the salt movement into the plant (Krishnamurthy et al., 2011, 2014a). Hence, we should also study the kinetics and dynamics of salt uptake or salt exclusion in roots as well as the histology of the roots in both tolerant and susceptible date palm varieties.

With trends leading to a greater production of date palm trees with enhanced salinity adaptive traits, the knowledge generated by Next Generation OMICS technologies should lead to a better understanding of the salt-adaptation mechanisms in the date palm. Thus, comparative RNAseq, differential proteomics and metabolomics, and the use of biochemical tools to understand the metabolic pathways associated with enhanced tolerance may lead to the discovery of novel gene(s) and metabolic pathways associated with salt-adaptation in date palms. Heterologous expression analysis in model plants could be used for the subsequent functional characterization of candidate genes. The generation of knockout mutants in the date palm would allow the application of reverse genetics to identify and characterize the function of unknown genes in the long term.

Several genome and transcriptome projects have been accomplished in the past to identify key genes for salt tolerance in various plant species. Unfortunately, the outcomes of these projects were rather modest because adaptation to salt is a multigenic trait in plants (Wang et al., 2012; Leonforte et al., 2013). A recent proteomic analysis of membrane proteins from the mangrove tree *Avicennia officinalis* helped to identify numerous membrane transporters and other membrane-integral proteins (Krishnamurthy et al., 2014b). However, on its own, this study was unable to provide a mechanistic basis for the salt tolerance exhibited by the mangroves, and it can only serve as a basis for future investigations into the mechanism.

Reverse genetics, which is based on loss- and gain-of-gene function principles, is a powerful method in functional genomics research (Colbert et al., 2001). This tool can also be used to study salinity tolerance in the date palm. Various strategies to generate random point mutations, such as ethyl methane sulfonate (EMS) and ionizing radiation treatments and the use of RNAi, transposon and T-DNA mutagenesis approaches, help identify genes associated with salinity tolerance based on the phenotype of the screened mutants. This technology, coupled with the next generation sequencing (NGS) tools, will allow multiplexing of gene targets thereby providing a greater chance of locating the target genes. However, these strategies require the establishment of an efficient propagation and *Agrobacterium*-mediated genetic transformation protocols for the date palm (Mousavi et al., 2014).

Taken together, this suggests that deciphering salt-adaptation mechanisms based on the variation in a single cellular product category (i.e., transcript or protein abundance in isolation) between salt-treated and untreated seedlings is unlikely to generate enough information that provides a more complete picture. However, the use of multi-disciplinary techniques (and multi-OMICS tools) to analyze various cellular products may provide a higher possibility of detecting additional elements associated with this phenotype in the date palm. Such an approach will undoubtedly make the genetic manipulation process, which aims to further improve salt-adaptation, more objective and efficient. For example, the information obtained from digital gene expression (DGE) analysis alone may not lead to the identification of key genes and mechanisms associated with salinity adaptation capacity in the date palm. However, combining that study with a global proteomics and hormonal analysis may lead to the discovery of critical pieces of the puzzle in this mechanism. It is worth highlighting in this context that studying the posttranslational modifications of membrane transporter proteins is a priority since these proteins may not vary in their amounts, but their levels of posttranslational modifications, such as phosphorylation in response to saline conditions, may vary significantly. Being a complex trait at both the genetic and physiological levels, such experiments may deliver important information that can be adopted by plant breeders to improve salinity tolerance in the date palm. Again, this may be achieved by transgenic means or by generating genetic crosses when the genes are present in closely related species or varieties. Consequently, the discovery of salt tolerant gene(s) will help breeders to select parent varieties (germplasm) and progenies using the marker-assisted selection strategy.

The outcome of the physiological and multi-OMICS analyses may provide answers to the following fundamental questions:

(1) What is the most salt-adaptive date palm variety?
(2) What is (are) the mechanism(s) behind salt-adaptation in the date palm tree?
(3) Which genes are responsible for the salt-adaptation in roots and leaves?
(4) Is there a crosstalk between the genetic network in roots and leaves during the adaptation procedure?
(5) What are their gene products and their effects on the phenotype?
(6) What are the posttranslational modifications associated with salinity tolerance in the date palm?

## Obstacles Facing Salt Tolerance Research in the Date Palm

There are hundreds of different varieties of date palm with distinct names; however, only a few of these are dominant and are being actively cultivated (Krueger, 1998). The nomenclature as well as the genetic makeup of date palm varieties varies from one country to another. This is a confusing situation that leads to complications. Therefore, worldwide nomenclature standardization is a necessity for successful date palm research. This should be accompanied by the establishment of an international germplasm bank through which defined germplasm can be accessible. This will facilitate seed exchange between laboratories and will enable various research lines in date palm, including salt tolerance research.

Date palms have a long juvenile period with the minimum generation time (from seed to seed) ranging from 5 to 7 years. Therefore, any classical genetic breeding or quantitative genetic program (Fan et al., 2015) aiming to decipher and enhance salinity tolerance is a time-consuming process. However, the use of modern biotechnological tools in this respect involving direct genetic manipulation, such as genetic engineering, may offer suitable solutions. Experiments aimed at salinity tolerance require the use of plants with well-defined genetic backgrounds. Such plants will have relatively uniform developmental stages, which will help to obtain reliable results. Therefore, we maintain that the use of genetically divergent adult trees or asexually reproduced basal offshoots growing in the field (whose growth is not under controlled conditions) and used to carry out differential experiments is unsatisfactory.

Date palm is a perennial dioecious fruit plant. Fruits are normally produced by cross-pollination with pollen grains acquired from male flowers of different date palm sources (Zaid and de Wet, 2002b). Farmers often select pollen grains for the pollination process based on the availability and the compatibility of the pollen grains with various varieties during a certain time of the season. Therefore, screening for salt tolerance using seeds of different varieties is also not technically acceptable because of the possible difference in paternal genetic background of the same variety. Under controlled conditions, this problem can be resolved by using a uniform paternal genetic background. In this strategy, flowers of different varieties can be pollinated using a common source of pollen grains. However, this approach does have some limitations; for example, female and male varieties may flower at different times or may not be compatible.

Another source of genetically homogenous varieties could be tissue culture-derived date palm plantlets (Zaid and de Wet, 2002c). However, this strategy has its own limitations. One such constraint is the availability of a wide range of date palm varieties in the commercial tissue culture laboratories. Other restrictions include the presence of somatic variations between plantlets of the same variety and the possible malformation of root systems due to the use of excessive amounts of hormones during the tissue culture process. These abnormalities may affect the subsequent physiological and molecular results.

In conclusion, despite being one of the oldest cultivated trees (Nixon, 1951; Zohary and Hopf, 2000), only a relatively small amount of research has been done regarding salinity adaptation mechanisms in the date palm. Research aimed at achieving a better understanding of this mechanism represents a golden opportunity for scientists to decode a black box of genetic resources that have evolved and developed in severe abiotic stress conditions for a super-tolerant phenotype. Since conventional breeding of the date palm is an arduous procedure, scientists should take advantage of the recent advances in Next Generation OMICS technologies. Complementary research should focus on the physiological changes associated with salinity tolerance. Together, information obtained from these research lines may provide a comprehensive view of salinity tolerance in date palms and facilitate the development of applications toward improving the salinity tolerance of this important crop.

### Conflict of Interest Statement

The authors declare that the research was conducted in the absence of any commercial or financial relationships that could be construed as a potential conflict of interest.
